# FcγRIIB mediates antigen-independent inhibition on human B lymphocytes through Btk and p38 MAPK

**DOI:** 10.1186/s12929-015-0200-9

**Published:** 2015-10-16

**Authors:** Shiang-Jong Tzeng, Wan-Yu Li, Hui-Ying Wang

**Affiliations:** Graduate Institute of Pharmacology, College of Medicine, National Taiwan University, Room 1118, No.1, Section 1, Ren-Ai Road, Taipei, 10051 Taiwan

**Keywords:** FcγRIIB, Antigen, Human B cell, Btk, p38 MAPK

## Abstract

**Background:**

The inhibitory Fc receptor, FcγRIIB, has emerged as a key negative regulator of B cell activation and as such is predicted to play an essential role in controlling antibody-mediated autoimmune diseases in humans. Recent studies have shown that crosslinking the FcγRIIB independently of the B-cell receptor (BCR) results in apoptosis in both mouse and chicken B cells. However, the human B cell subpopulations that are susceptible to BCR-independent, FcγRIIB-mediated regulation are not known. How FcγRIIB mediates this inhibition to affect B cell homeostasis is also not determined.

**Results:**

We isolated naïve B cells, memory B cells and plasma cells (PCs) from peripheral blood of healthy donors and used differentiated PCs in culture to examine the effects on them by FcγRIIB crosslinking. We showed that human PCs, memory and naïve B cells all expressed FcγRIIB with expression on PCs being the highest in circulation. Moreover, they were sensitive to direct inhibition by FcγRIIB through Btk and p38 MAPK. Similarly, PCs resulting from the antigen-independent differentiation of memory B cells *in vitro* were inhibited by FcγRIIB cross-linking but memory B cell activation itself, as measured by proliferation, was unaffected. In contrast, both the proliferation and differentiation of naïve B cells to PCs were blocked by FcγRIIB crosslinking.

**Conclusion:**

These results suggest a mechanism to control antibody levels involving the differential expression of FcγRIIB on B cell subpopulations, in which the FcγRIIB functions independently of the BCR to eliminate antibody-secreting effector cells and inhibit naïve B cell proliferation without compromising the long-lived antigen-specific memory B cells. Importantly, FcγRIIB requires Btk and p38 MAPK to mediate antigen-independent inhibition in human B cells. Taken together, our data underscore the importance of antigen-independent inhibition by FcγRIIB in the prevention from antibody-mediated autoimmune diseases and in the regulation of B cell homeostasis.

## Background

The low affinity Fc receptor, FcγRIIB, is a potent B-cell inhibitory receptor and as such plays a central role in controlling antibody-mediated autoimmunity [1, 2]. In humans, mutations in FcγRIIB have been associated with systemic lupus erythematosus (SLE) [3, 4], and memory B cells and PCs in individuals with SLE express lower levels of FcγRIIB as compared to memory B cells and PCs in healthy individuals [5], a factor that is suggested to contribute to disease [6]. The FcγRIIB exerts its inhibitory effect on B cells via what appears to be two distinct signaling pathways. When coligated to the B-cell receptor (BCR) through the binding of antigen-containing immune complexes (ICs), the FcγRIIB inhibits antigen-specific antibody responses by blocking BCR signaling. Such inhibition involves the phosphorylation of the immunoregulatory tyrosine inhibitory motif (ITIM) in the cytoplasmic domain of the FcγRIIB by Lyn, and the recruitment of the lipid phosphatase, SHIP [7–9]. In contrast, recent evidence indicates that when clustered independently of the BCR, the FcγRIIB initiates an ITIM-, Lyn- and SHIP-independent pathway that triggers apoptosis through a mechanism that involves c-Abl family kinases [10, 11]. Thus, the FcγRIIB has the ability to block both the BCR-dependent, antigen-driven activation of B cells as well as antigen-independent, BCR-independent B cell activation. There is considerable evidence that the BCR-dependent FcγRIIB inhibitory pathway plays an important role in regulating the antigen-driven activation of naïve B cells to proliferate and differentiate to PCs [12]. However, the B cell subpopulations that are susceptible to BCR-independent, FcγRIIB-mediated inhibition are not as clearly delineated. It was recently shown, in mice, that long-lived bone marrow PCs express the FcγRIIB and that engaging the FcγRIIB by ICs induces these bone marrow PCs to undergo antigen-independent apoptosis [13]. In addition, Rahman *et al.* [14] provided evidence, in mice, that the FcγRIIB regulates PCs but not germinal center B cells. Thus, in mice, the accumulation and persistence of PCs in the bone marrow appears to be regulated by ICs through the inhibitory FcγRIIB independently of the BCR. At present, the effect of FcγRIIB crosslinking on the antigen-independent activation of human B cell subpopulations is not known.

Here we investigate the ability of the BCR-independent FcγRIIB inhibitory pathway to directly inhibit human peripheral blood PCs and to block the antigen-independent activation of human naïve and memory B cells to proliferate and differentiate into PCs *in vitro*. We provide evidence that the FcγRIIB is most highly expressed on PCs and that IgG-secreting cells from human peripheral blood are sensitive to direct inhibition by FcγRIIB-crosslinking. Crosslinking the FcγRIIB also inhibits PCs resulting from the antigen-independent differentiation of human memory B cells but has no direct effect on the antigen-independent activation of memory B cells to proliferate. Lastly, we show that both the antigen-independent proliferation and differentiation of naïve B cells *in vitro* are blocked by FcγRIIB crosslinking. Taken together, these results suggest that the BCR-independent FcγRIIB signaling pathway may play an important role in humans in acutely controlling antibody levels by inhibiting antibody-secreting PCs and the activation of naïve B cells without affecting the long-lived memory B-cell pool, which is capable to quickly expand and differentiate into PCs to provide protective humoral immunity upon re-encountering antigen.

## Methods

### Antibodies and reagents

The FcγRIIB-specific mAb AT10 (biotinylated, FITC- and PE-conjugated) was obtained from Abcam (Cambridge, MA, USA) [15]. Goat IgG and rabbit anti-goat IgG were used to make ICs as previously described [11]. Mouse IgG1, rabbit peroxidase-anti-peroxidase (PAP) ICs were purchased from Jackson ImmunoResearch Laboratories (West Grove, PA, USA). CpG 2006 was purchased from Santa Cruz Biotechnology (Dallas, TX, USA). Mouse isotype control mAbs and mAbs specific for CD19 (SJ25C1), CD45 (HI30), CD27 (L128), CD38 (HB7) and CD14 (M5E2) were purchased from BD Biosciences (San Jose, CA, USA). Recombinant human IL-21, IL-2 and IL-10 and human sCD40L were purchased from PeproTech (Rocky Hill, NJ, USA). Antibodies specific for CD27 (O324), CD19 (HIB19) and CD20 (2H7) were purchased from eBioscience (San Diego, CA, USA). Human B cell isolation kit was obtained from BD Biosciences. *Staphylococcus aureus* Cowan (SAC) and lectin from Phytolacca Americana (Pokeweed mitogen, PWM) were obtained from Merck Millipore (Billerica, MA, USA) and Sigma-Aldrich (St. Louis, MO, USA), respectively. Carboxyfluorescein succinimidyl ester (CFSE) was acquired from eBioscience (San Diego, CA, USA). SB203580, SP600125, Z-VAD-FMK, LFM-A13 and ibrutinib (PCI-32765) were all purchased from Selleck Chemicals (Houston, TX, USA).

### Isolation and culture of human peripheral blood B cells

Human peripheral blood was obtained from healthy donors with informed consent and the use of it was conformed to the approved guidelines established by the Institutional Review Board of National Taiwan University Hospital (reference numbers: 201005012R and 201307019RINB). Erythrocytes in human peripheral blood cells were first depleted by lysis buffer (150 mM NH_4_Cl, 10 mM KHCO_3_, 1 mM EDTA, pH 7.4). After centrifugation the pellets were layered over a Ficoll-Paque Plus (GE Healthcare, Uppsala, Sweden) gradient (2,000 rpm, 20 min) to collect lymphocytes at the gradient interface. For flow cytometric analysis cells were further layered over a fetal calf serum gradient to remove platelets (800 rpm, 15 min) to decrease non-specific binding to mAbs. The cell pellet was washed, resuspended and cultured on plastic cell-culture dishes for 30–60 min to remove adherent cells. Non-adherent cells were harvested and resuspended in PBS (0.5 % BSA) and B cells were purified by negative selection using the human B cell isolation kit (Merck Millipore) according to the manufacturer’s protocol. Biotinylated mouse mAbs specific for CD3 and CD16 (BD Biosciences) were added to the cocktail mAbs to increase the efficiency of depleting non-B cells. The resulting B cell populations were 95–98 % pure as assessed by CD19 and/or CD20 expression. Purified B cells were further separated into CD27^+^ memory and CD27^−^ naïve subsets using CD27 mAb-conjugated microbeads (Miltenyi Biotec). Alternatively naïve and memory B cells were obtained by cell sorting gating on CD19^+^ cells and then sorting into CD27^+^ and CD27^−^ populations using FACSAria cell-sorting system (BD Biosciences). Likewise, PCs were sorted as CD19^+^CD27^+^CD38^+^. The experimental results from B cells fractionated by mAb-conjugated beads or by sorting were comparable. Purified cell populations (5–10 × 10^5^/ml) were cultured in RPMI-1640 medium (Thermo Scientific) supplemented with 10 % heat-inactivated fetal calf serum, 2 mM L-glutamine, 100 U/ml penicillin, 100 mg/ml streptomycin and 50 μM β-mercaptoethanol. CpG was added at the concentration of 5 μg/10^6^ cells/ml. SAC (at a 1: 10,000 dilution), PWM (at a 1:100,000 dilution), recombinant human IL-2 (10 ng/ml), IL-10 (10 ng/ml), IL-21 (100 ng/ml) and sCD40L (1 μg/ml) were added accordingly [16–22].

### Flow cytometry

To detect FcγRIIB surface expression, cells were incubated with AT10-FITC. The percentage of dead cells was measured by 7-aminoactinomycin D (7-AAD). Multi-color flow cytometry was performed using Fortessa (BD Biosciences) and analyzed using Flowjo v7.6 (Tristar).

### Proliferation and cell division assays

Purified B cells (10^6^/ml) were stimulated in triplicates in 96-well plates in the presence of an isotype control mAb or AT10 mAb (5 μg/ml) for 48–72 h before adding [^3^H] methyl thymidine (1 μCi/well) for an additional 16 h. Cells were harvested and transferred onto a 96-well filter paper (PerkinElmer-Wallac, Waltham, MA, USA) and the radioactivity associated with the filter papers was determined using a β counter. CFSE labeling to detect dividing cells was performed following the manufacturer’s protocol (eBioscience).

### ELISPOT assay

Ninety six-well MultiScreen HTS filter plates (Merck Millipore) were coated with F (ab’)_2_ goat antibodies specific for human IgG + IgM antibodies (5 μg/ml) in PBS at 4 °C overnight. After washing with PBS and blocking with culture medium, 100 μl fresh medium was added to each well before adding cells (2-fold serial diluted) for culture at 37 °C for 4–6 h. Plates were washed with PBS (0.1 % Tween 20) and then incubated in 100 μl/well PBS (0.5 % BSA) with biotinylated F (ab’)_2_ goat antibodies specific for human μ chain or γ chain (1:5000, Jackson ImmunoResearch Laboratories) at 4 °C overnight. After washing, alkaline phosphatase-conjugated streptavidin (Merck Millipore) was added (100 μl/well) and incubated at ambient temperature for 2–4 h and the plates were developed using ACE kit (Merck Millipore). After drying, the plates were scanned and analyzed using C.T.L. ImmunoSpot scanner and software (version 4).

### Statistical analyses

Graphs and histograms were plotted using GraphPad Prism 5 (San Diego, CA, USA). Statistical analysis was performed using one-way ANOVA (repeated measures for paired values) and Turkey’s multiple comparison tests.

## Results

### The expression of FcγRIIB on peripheral blood PCs, memory and naive B cells

**Fig. 1:**
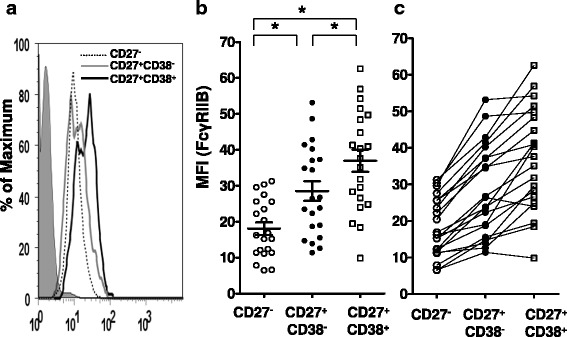
Human memory B cells, naïve B cells and PCs express FcγRIIB. **a** B cells were first gated by CD45^+^CD19^+^ and further gated to CD27^−^, CD27^+^CD38^−^ and CD27^+^CD38^+^ subsets for their expression levels of FcγRIIB . A representative histogram of the mean fluorescence intensity (MFI) of one donor is shown. **b** A scatter dot plot (mean ± SEM) of the expression levels of FcγRIIB of the three subpopultions of B cells from 21 donors is shown. The asterisks indicated that the comparisons are statistically significant (*P* < 0.01). **c** The FcγRIIB expression levels of memory B cells, naïve B cells and PCs for individual donors are shown

To determine the populations of human peripheral B cells that are potential targets of FcγRIIB-mediated inhibition, we determined the expression of FcγRIIB on naïve B cells (CD19^+^CD27^−^CD38^−^), memory B cells (CD19^+^CD27^+^CD38^−^) and PCs (CD19^+^CD27^+^CD38^+^) by flow cytometry (Fig. 1). The three subpopulations showed a hierarchical order of FcγRIIB expression with PCs > memory B cells > naïve B cells (Fig. 1a, b). This pattern held in the vast majority of cases when the expression of FcγRIIB was compared between the B cell populations within each individual donor (Fig. 1c). These findings suggest that each of these human B cell populations is potentially susceptible to FcγRIIB regulation and that the outcome of FcγRIIB-crosslinking may differ between the populations, either quantitatively or qualitatively, as a consequence of the differences in the levels of FcγRIIB expression.

### FcγRIIB crosslinking inhibits viability of CD19^+^CD27^+^CD38^+^ PCs and *in vitro* differentiation of PCs

**Fig. 2:**
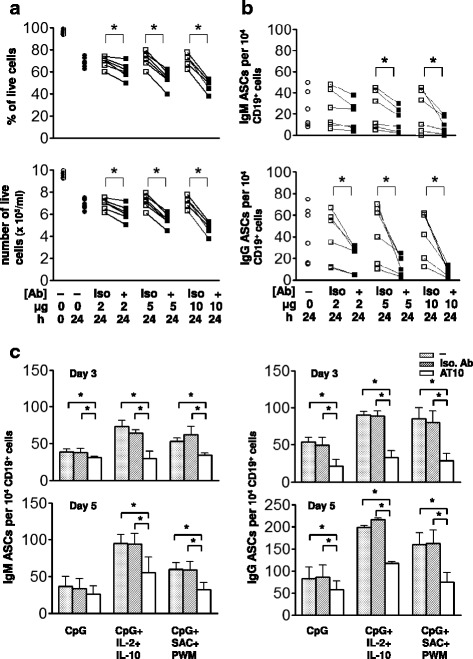
Crosslinking FcγRIIB inhibits cell viability of purified primary PCs and subsequent PC differentiation *in vitro*. **a** Purified PCs (10^6^/ml) were cultured for 24 h in the absence (circle) or presence of various doses (2–10 μg/ml) of isotype (squares) or AT10 (filled squares) mAbs. The number and percentage of dead cells were determined by staining with 7-AAD and side scattering using flow cytometry. **b** Primarily isolated CD19^+^ B cells (10^6^/ml) were cultured for 24 h as in (**a**) in 96-well ELISPOT plates as described in Methods. **c** Purified B cells from 6 donors were cultured as in (**b**) with the addition of CpG (5 μg/ml) alone, CpG + IL-2 + IL-10 or CpG + SAC + PWM for three or five days without antibody or in the presence of an isotype matched control mAb or AT10 mAb (5 μg/ml). The asterisks indicate that the differences between the groups compared are statistically significant (*P* < 0.05)

To determine the direct effect of FcγRIIB crosslinking on ASCs, CD19^+^CD27^+^CD38^+^ PCs were isolated from human peripheral blood cells as described in Material and Methods. Taken directly from the peripheral blood the purified PCs gradually became quiescent and ~30 % of them underwent spontaneous apoptosis in culture medium after 24 h. Nevertheless, incubation of these PCs for 24 h with the presence of FcγRIIB-specific mAbs AT10, or ICs to crosslink the FcγRIIB resulted in a significant reduction of ~15–20 % in cell viability (Fig. 2a). This circulating population of PCs appeared to contain mostly IgG-ASCs in a frequency of ~50–100 per 10^4^ CD19^+^ cells, and many fewer IgM-ASCs (0–50 per 10^4^). Crosslinking the FcγRIIB also significantly reduced the number of both IgM- and IgG-ASCs (Fig. 2b). Shown were the percent of viable cells and the numbers of IgG- and IgM-secreting cells 24 h after treatment with AT10 mAb for six different individuals. Thus, regardless of their BCR specificities the ASCs from peripheral blood are sensitive to direct inhibition by FcγRIIB crosslinking. Since no exogenous growth factors or cytokines were added into the medium, this inhibitory effect can be attributed to an increase in apoptosis rather than a block in proliferation as there was no appreciable change in cell number in either control or treated cultures (Fig. 2a). Consistently, the doubling time of human primary B cells in culture was reported to be ~36 h [23], which was longer than the period of 24 h to observe the effect of AT10 mAb (Fig. 2a and b).

We also examined the effect of FcγRIIB crosslinking on the differentiation of ASCs from B cells *in vitro*. Recent evidence indicated that human peripheral blood B cells express Toll-like receptor 9 (TLR9) and are responsive to TLR9 agonist, CpG ODN, to differentiate into CD19^+^CD27^+^CD38^+^ PCs in 3 to 5 days *in vitro* [23, 24]. Consistent with previous findings, we observed that most (~80–90 %) mature circulating antibody-secreting PCs died during the first 48 h of culture [23, 24]. Newly differentiated IgM- and IgG-secreting PCs appeared by day three in culture at a frequency of approximately 50 PCs per 10^4^ CD19^+^cells (Fig. 2c) and by day five in culture the number of IgG-antibody secreting PCs increased to approximately 100 per 10^4^ CD19^+^ cells (Fig. 2c). The presence of the AT10 mAb or ICs significantly reduced the number of both IgM- and IgG-ASCs that resulted from CpG stimulation of PCs measured after 3 days in culture (Fig. 2c). The addition of IL-2 and IL-10 or SAC and PWM that are stronger inducers to BCR to the CpG-containing cultures increased the resulting number of both IgM- and IgG-ASCs [16] and in both cases the number of ASCs was reduced significantly by the presence of the AT10 mAb but not by an isotype matched control mAb (Fig. 2c). Taken together these results indicate that the human ASCs either isolated directly from human peripheral blood or resulting from the differentiation of PCs *in vitro* are sensitive to inhibition by FcγRIIB crosslinking independent of BCR.

### The effect of FcγRIIB crosslinking on the antigen-independent differentiation of B cells to PCs

**Fig. 3:**
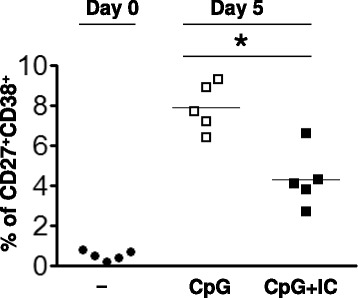
FcγRIIB crosslinking reduces the number of CD27^+^CD38^+^ purified PCs that differentiate from BCR-independent activation of B cells. Purified B cells (10^5^/ml) were cultured with CpG (5 μg/ml) in the absence or presence of rabbit PAP ICs (5 μg/ml) for five days. The percentage of CD27^+/hi^CD38^+^ cells by flow cytometry is shown as a histogram before and after the 5-day culture from five donors. The asterisk indicates statistical significance of the difference between the two groups compared (*P* < 0.05)

Human B cells have been shown to respond to the TLR9 agonist, CpG, by proliferating and differentiating into antibody secreting PCs. Bernasconi *et al.* [17, 18] reported that CpG single agent activated IgM memory B cells (CD19^+^CD27^+^IgG^−^) and switched memory B cells (CD19^+^CD27^+^IgD^−^IgM^−^) to proliferate and differentiate into antibody secreting PCs, but had little effect on naïve B cells (CD19^+^CD27^−^). We determined the effect of crosslinking the FcγRIIB on the differentiation of purified CD19^+^ peripheral blood B cells to PCs in response to CpG, using the ICs. The highly purified CD19^+^ peripheral blood B-cell population contained less than 0.5 % CD38^+^ PCs by flow cytometry (Fig. 3) and no ASCs by ELISPOT analyses after 3 days in culture (data not shown). Culturing these purified B cells with CpG induced the differentiation to CD19^+^CD27^+^CD38^+^ PCs over a 5-day culture as measured by flow cytometry (Fig. 3). In the absence of CpG no PCs were detected in the 5-day cultures (data not shown). In the presence of CpG, 7.9 to 8.1 % of the cells recovered from the 5 day cultures were PCs and this number was significantly decreased to 3.5 to 4.2 % by the presence of the AT10 mAb or ICs in the 5-day cultures. An isotype matched control mAb had no effect on the CpG-induced generation of PCs. Consistently, in the presence of CpG the reduced number of PCs by AT10 mAb or ICs appeared to correlate to the decrease of ASCs (Fig. 2c and Fig. 3).

**Fig. 4:**
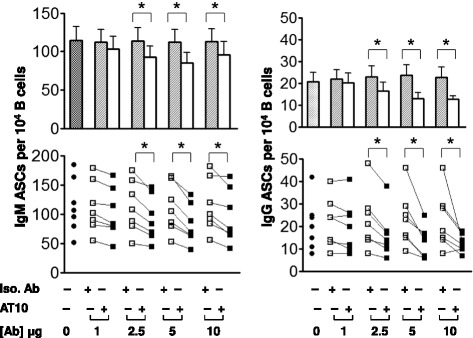
FcγRIIB crosslinking reduces the number of antibody-secreting PCs resulting from BCR-independent activation of B cells. Purified B cells were treated with CpG (5 μg/ml) without additional antibody or in the presence of an isotype matched control mAb or AT10 mAb in graded concentrations (0, 1, 2.5, 5 or 10 μg/ml) for five days. ELISPOT analyses were performed to measure total IgM- and IgG-antibody-secreting PCs. Data are shown as an average of all individuals analyzed (top) or for individual donors (bottom). The asterisks indicate that the differences between the groups compared are statistically significant (*P* < 0.05)

The effect of crosslinking the FcγRIIB on the differentiation of CD19^+^ B cells in response to CpG was also determined by an ELISPOT assay, capturing antibodies secreted by PCs using either IgM- or IgG-specific antibodies. CpG induced the differentiation of both IgM- and IgG-secreting PCs, suggesting that both IgM memory B cells and switched memory B cells were activated to differentiate to PCs. In the absence of CpG few viable cells and no antibody secreting PCs were recovered from the 5-day cultures (data not shown). The addition of the AT10 mAb reduced the numbers of both IgG- and IgM-secreting PCs in cultures in a dose-dependent fashion as compared to an isotype matched nonspecific control mAb (Fig. 4). The average of the results from B cells purified from several individual donors showed that crosslinking the FcγRIIB resulted in a significant decrease in the number of IgM- and IgG-secreting PCs (Fig. 4, top). An analysis of each individual donor showed a large individual to individual variation in the number of PCs that resulted from CpG stimulation of B cells but for each individual crosslinking the FcγRIIB reduced that number significantly (Fig. 4, bottom). FcγRIIB crosslinking did not have a significant effect on the overall cell numbers or cell viability in culture (data not shown), suggesting that the effect of FcγRIIB crosslinking may be at the level of the relatively small population of newly generated PCs.

### FcγRIIB crosslinking reduces the number of PCs resulting from the differentiation of both naïve and memory B cells

**Fig. 5:**
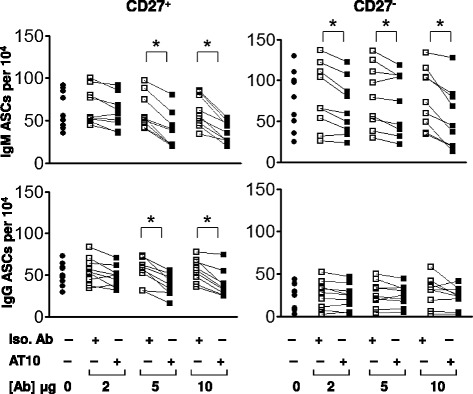
FcγRIIB crosslinking reduces the number PCs resulting from the BCR-independent differentiation of CD27^+^ memory and CD27^−^ naïve B cells. CD27^+^ and CD27^−^ B cells from eight donors were treated with CpG as in Fig. 4 at various concentrations of isotype matched control or AT10 mAbs (0, 2, 5, or 10 μg/ml) for five days. The number of IgM- and IgG-antibody-secreting PCs was determined by ELISPOT assay. The asterisks indicate that the differences between the groups compared are statistically significant (*P* < 0.05)

The effect of FcγRIIB crosslinking on the antigen-independent, CpG-induced differentiation of memory B cells and naïve B cells to PCs was tested. To do so, purified peripheral blood CD19^+^CD38^−^ B cells were separated into CD27^+^ memory and CD27^−^ naïve B cell. Memory and naïve B cells were incubated with CpG in the presence of graded concentrations of either AT10 mAb or an isotype matched control mAb (Fig. 5). CpG alone induced the differentiation of both IgG- and IgM-secreting PCs from memory B cells from the nine individual donors analyzed and the number of PCs was reduced by AT10 mAb in a dose dependent fashion but not by the isotype matched control mAb. The AT10 mAb was effective in reducing the number of IgM- and IgG-secreting PCs at 5 μg/ml. Naïve B cells were only weakly responsive to CpG as described previously by Bernasconi *et al.* [17, 18] and Jiang *et al*. [19] and most of the resulting PCs secreted IgM (Fig. 5). The number of IgM-secreting PCs was reduced by the AT10 mAb with the largest decrease observed at 10 μg of AT10.

**Fig. 6:**
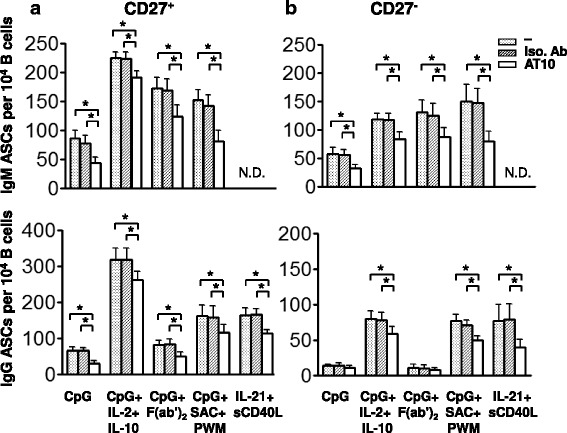
FcγRIIB crosslinking reduces the number of PCs that result from the differentiation from CD27^+^ memory and from CD27^−^ naïve B cells to a variety of stimuli. **a** CD27^+^ memory B cells or **b** CD27^−^ naïve B cells were incubated without additional antibody or in the presence of an isotype matched control mAb or AT10 mAb (5 μg/ml) and either CpG (5 μg/ml) alone, CpG + IL-2 + IL-10, CpG + F (ab’)_2_ fragment of anti-Ig, CpG + SAC + PWM or IL-21 + sCD40L. The number of IgM- and IgG-antibody-secreting PCs was determined by ELISPOT assay. The asterisks indicate that the differences between the groups compared are statistically significant (*P* < 0.05)

We also tested the ability of the FcγRIIB to reduce the number of PCs resulting from the response of B cells to combinations of CpG and other mitogens, cytokines and BCR agonists that have been reported to influence the response of naïve and memory B cells to CpG (Fig. 6). Consistent with previously reported by Jiang *et al*. [19], CpG alone induced naïve B cells into more IgM-ASCs than IgG-ASCs in a 5-day culture (Fig. 6). Bernasconi *et al.* showed that the addition of the cytokines IL-2 and IL-10 to the CpG-containing cultures increased the numbers of PCs produced from memory B cells and also resulted in a limited differentiation of naïve B cells [18, 20]. The addition of IL-2 and IL-10 to CpG-containing cultures significantly increased the number of both IgM- and IgG-secreting PCs and this number was reduced by the AT10 mAb. We also tested the effect of F (ab’)_2_ anti-Ig in combination with CpG on the differentiation of memory B cells as Bernasconi *et al.* had shown that BCR crosslinking influenced the response of IgM memory and naïve cells to CpG [18]. Incubating memory B cells with CpG and F (ab’)_2_ anti-Ig resulted in an increase in the IgM-secreting PCs as compared to CpG alone but had no effect on the IgG-secreting PCs, suggesting that crosslinking the BCR only affected the activation of IgM memory B cells. Crosslinking the FcγRIIB decreased the number of PCs under these conditions. Crotty *et al.* [21] showed that a combination of CpG and *S. aureus* Cowan (SAC) and Pokeweed mitogen (PWM) activated memory B cells (CD19^+^CD27^+^) to differentiate to IgG secreting PCs. Culturing memory B cells with CpG in combination with SAC and PWM induced an increase in both IgM- and IgG-producing PCs over CpG alone and the AT10 mAb decreased the number of PCs in each case. Lastly, a combination of IL-21 and sCD40L induced memory B cell differentiation exclusively to IgG-secreting PCs [22] and the number of IgG-secreting PCs was blocked by FcγRIIB crosslinking. These findings indicate that the antigen-independent activation of memory B cells to differentiate to PCs by a variety of different stimuli is inhibited by FcγRIIB crosslinking. Given that PCs are themselves inhibited by FcγRIIB crosslinking, the simplest interpretation of these results is that the FcγRIIB acts to inhibit the newly generated PCs rather than blocking the activation of the memory B cells themselves.

Naïve B cells differentiated primarily into IgM-secreting PCs in response to all stimuli tested [18] with the exception of IL-21 and sCD40L that promotes isotype switching [22] and in each case the number of PCs was decreased in the presence of AT10 mAb but not an isotype matched control mAb (Fig. 6). The effect of AT10 mAb was also tested on PCs differentiated by IL-21 in combination with sCD40L, conditions that induced isotype switching in B cells [22]. These culture conditions yielded exclusively IgG-secreting PCs and these IgG-ASCs were reduced by the AT10 mAb but not the isotype matched control mAb. These results suggest that the PCs differentiating from naïve B cells are inhibited by FcγRIIB crosslinking.

### FcγRIIB crosslinking triggers apoptosis of human B cells through Btk and p38 MAPK

**Fig. 7:**
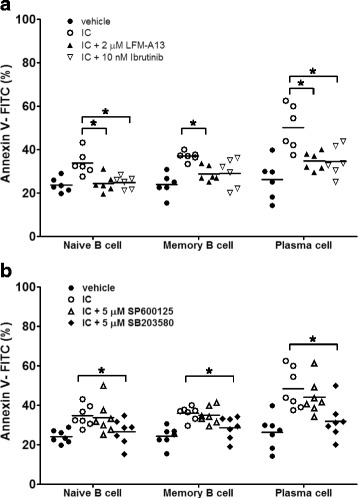
Crosslinking FcγRIIB triggers apoptosis through Btk and p38 MAPK in human B cells. **a** Purified naïve, memory B cells and PCs (10^5^/ml) from seven donors were each treated with 10 μg/ml of ICs in the presence of either 2 μM of LFM-A13 or 10 nM of ibrutinib for 24 h. Apoptotic cells were determined by Annexin V staining (Biolegend) using flow cytometry. **b** Three subsets of purified human B cells were treated separately in the absence or presence of 10 μg/ml ICs with addition of either 5 μM of SP600125 or 5 μM of SB203580 for 24 h. Apoptotic cells were measured and analyzed as in **a**. The asterisks indicate that the differences between the groups compared are statistically significant (*P* < 0.05)

We next investigated the molecular mechanism for the antigen-independent apoptosis induced by FcγRIIB. Since Pearse *et al.* reported that FcγRIIB crosslinking triggers apoptosis through Btk and JNK in chicken DT40 B cells [10], we first examined this possibility with two selective Btk inhibitors, LFM-A13 and ibrutinib, both of which significantly blocked apoptosis induced by ICs to a similar degree compared to controls. The reversal of FcγRIIB-mediated inhibition was most apparent in PCs (Fig. 7a). This is consistent with the fact that PCs have the highest expression level of FcγRIIB, thereby conferring the highest induction of apoptosis among circulating B cells (Figs. 1 and 2). To further investigate the signaling intermediaries downstream of Btk, we treated naïve, memory B cells and PCs with either SP600125, a JNK inhibitor or SB203580, a p38 MAPK inhibitor in the presence of ICs for 24 h. Unlike previously reported in DT40 chicken B cells [10], the p38 MAPK, but not JNK was involved in FcγRIIB-mediated apoptosis in human B cells (Fig. 7b).

### The effect of crosslinking the FcγRIIB on naïve and memory B cell proliferation

**Fig. 8:**
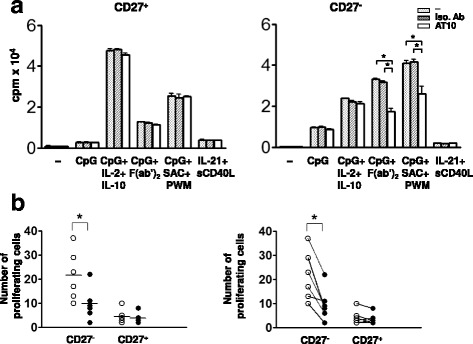
FcγRIIB inhibits the proliferation of CD27^−^ naïve B cells but not CD27 ^+^ memory B cells. **a** CD27^+^ and CD27^−^ B cells (10^6^/ml) were cultured for two days before the addition of ^3^[H] methyl thymidine for another 16 h. Antibodies (5 μg/ml) and stimuli were added to cells in the beginning of culture as in Fig. 6. A representative histogram of more than 3 donors was shown. The asterisks indicate that the differences between the groups compared are statistically significant (*P* < 0.05). Similar results were obtained when cells were cultured for 1–2 more days before adding ^3^[H] methyl thymidine. **b** CD27^+^ and CD27^−^ B cells (10^5^/ml) from six donors were culture for 48 h in the presence of 2 μg/ml of F (ab’)_2_ anti-Ig (IgM + IgG + IgA, Jackson Immunoresesearch) with (open circle) or without (closed circle) 10 μg/ml of ICs. Cells with decreased green fluorescence, indicative of cell division, were counted using flow cytometry and the absolute number (× 10^2^) was plotted as the Y-axis. The horizontal bar in each group represents mean of subjects and the asterisks indicate that the differences between the groups compared are statistically significant (*P* < 0.05)

We tested the effects of crosslinking the FcγRIIB on the proliferative responses of CD19^+^CD27^+^CD38^−^ memory B cells and CD19^+^CD27^−^CD38^−^ naïve B cells to the same combinations of CpG, cytokines and BCR agonists assessed above in Fig. 6. Memory B cells proliferated over a 2-day period to CpG in combination with IL-2 plus IL-10, F (ab’)_2_ anti-Ig or SAC plus PWM (Fig. 8a). The response in each case was refractory to the effects of the AT10 mAb even at high antibody concentrations (10 μg/ml) (data not shown). Moreover, in the presence of F (ab’)_2_ anti-Ig to crosslink BCR but absence of any differentiation agents assessed above, memory B cells showed no appreciable inhibition by ICs, either (Fig. 8b). These findings are consistent with the interpretation of the results above, namely that the FcγRIIB does not act at the level of the activation of memory B cells, which are known to be quiescent, progenitor cell-like and less responsive to mitogenic stimuli [25], but rather inhibits the resulting PCs.

Naïve B cells proliferated over a 5-day period to varying degrees in response to CpG alone or in combination with SAC plus PWM, IL-2 plus IL-10 or F (ab’)_2_ anti-Ig. Treatment with CpG in combination with SAC and PWM or with CpG and F (ab’)_2_ anti-Ig resulted in the greatest proliferative responses and these were significantly blocked by the AT10 mAb as compared to the isotype matched control mAb (Fig. 8a). Likewise, naïve B cells proliferated in response to F (ab’)_2_ anti-Ig alone but this was inhibited by ICs (Fig. 8b). AT10 had little effect on the naïve B cell proliferative responses to CpG alone or to CpG in combination with IL-2 and IL-10. This is likely because CpG mainly promotes differentiation rather than proliferation of human B cells in culture [18]. The selective effect of FcγRIIB crosslinking on the responses of naïve B cells to the different stimuli may reflect differences in either the quality or magnitude of the response. Taken together these results provide evidence that memory B cell responses are refractory to FcγRIIB regulation in contrast to naïve B cell responses that are sensitive to FcγRIIB inhibition in proliferation.

## Discussion

The FcγRIIB has been shown to serve as a critical peripheral checkpoint to regulate the levels of antibody and results indicate that the FcγRIIB has the ability to inhibit B-cell responses independently of the BCR. Recent studies have clarified which B-cell subpopulations are susceptible to BCR-independent FcγRIIB regulation in mice. Xiang *et al.* [13] demonstrated that the FcγRIIB is expressed on long-lived PCs and that these cells undergo apoptosis following FcγRIIB engagement by ICs and Rahman *et al.* [14] showed that in mice PCs but not germinal center B cells are regulated by the FcγRIIB. These finding are important as they offer a mechanism by which immunization and the resulting formation of ICs can reduce the number of pre-existing PCs and thereby create space for new PCs in the limited microenvironments of the bone marrow that support PC survival. Here we show that the FcγRIIB is expressed on human peripheral blood PCs as well as on naïve and memory B cells and that crosslinking the FcγRIIB inhibits human ASCs isolated directly from peripheral blood as well as PCs that resulted from the antigen-independent differentiation of human memory B cells. We also demonstrate that the signals transduced by FcγRIIB to exert antigen-independent inhibition require Btk and p38 MAPK. Lastly, we show that both the antigen-independent proliferation and differentiation of naïve B cells *in vitro* are blocked by FcγRIIB crosslinking. Our studies suggest that the FcγRIIB may play an important role in humans in acutely controlling antibody levels by inhibiting antibody secreting PCs and the activation and proliferation of naïve B cells. These findings also underscore a crucial mechanism to control B cell homeostasis through the peripheral checkpoints on human PCs and naïve B cells by a negative feedback determined by circulating antibody levels.

Recently it has been shown that polyclonal stimulation through TLRs in the absence of antigen triggers memory B cells to expand and differentiate into PCs providing a mechanism by which a long-lived PC can be continually replenished throughout an individual’s lifetime [17]. However, with the continuous generation of antibody-secreting PCs it is not clear how the levels of antibodies are controlled after reaching sufficient or perhaps even dangerous levels. Our findings that FcγRIIB crosslinking inhibited PCs generated from memory B cells in culture but had no effect on memory B cell proliferation suggests a mechanism by which the FcγRIIB acutely regulate antibody levels without affecting the memory B-cell pool.

Here we also show that although naïve B cells, memory B cells and PCs all express FcγRIIB, they do so at different levels with the lowest expression of FcγRIIB on naïve B cells and the highest levels on PCs. In general, in immune cells the FcγRIIB is coupled with activating receptors to balance the outcome of responses to complex multiple receptor engagements. Thus, the different levels of the FcγRIIB on naïve B cells, memory B cells and PCs may relate to the function of the FcγRIIB in controlling the particular activating receptors expressed in each cell type. The relatively high FcγRIIB expression on PCs may play an important role in triggering apoptosis to achieve homeostatic control. Importantly, we demonstrate that the underlying mechanisms of antigen-independent apoptosis through FcγRIIB depend on Btk and p38 MAPK in the human B cells. Although we previously showed that c-Abl family kinases are essential for FcγRIIB-mediated apoptosis in mouse A20 B cells [11], the c-Abl’s substrate motif (Y^264^QRP) on mouse FcγRIIB appears not conserved in the counterpart of human receptor (Y^258^PEC). This explains no effect of imatinib, a c-Abl inhibitor, on FcγRIIB-mediated apoptosis in human B cells (data not shown). Meanwhile, this result raises an interesting question on how and why this divergence occurs during the evolution of adaptive immunity. One additional caveat is that although there is a ~10–15 % less of apoptosis induced by ICs (Fig. 2a) than that of chicken DT40 B cells transfected with the mouse FcγRIIB for overexpression [11], the magnitude in apoptosis induction of these two cell types is both significant. This is also consistent with the notion that apoptosis mediated by FcγRIIB depends on the signal strength as we previously proposed [11].

Recent studies in mice have underscored the importance of the level of FcγRIIB expression in B cells in the regulation of antibodies levels. FcγRIIB-deficient mice show an increased accumulation of Ig-secreting cells in the spleen and an enhanced antibody response [12, 24, 26] and eventually die of a lupus-like glomerulonephropathy. Lupus-prone mice often down-regulate the expression of FcγRIIB and the resulting defects can be reversed by increasing FcγRIIB expression levels in B cells [27]. In humans, Mackay *et al.* [6] showed that peripheral blood CD27^+^ memory B cells express higher levels of FcγRIIB as compared to naïve B cells but that this level of FcγRIIB is considerably decreased on memory B cells from SLE patients. In addition, Su *et al.* [5] provided evidence that the expression levels of the FcγRIIB were decreased in memory B cells and PCs in individuals with active SLE as compared to normal controls. The decreased levels of FcγRIIB correlated with decreased levels of FcγRIIB-mediated suppression of BCR-induced calcium responses demonstrating a link between the level of FcγRIIB expression in human B cells and inhibitory activity. Xiang *et al.* [13] also showed that a multiple myeloma cell line with low FcγRIIB expression levels was resistant to apoptosis and that the susceptibility was restored when FcγRIIB surface expression was increased, indicating a link between the surface expression levels of FcγRIIB and the functional outcomes of crosslinking. We show here that the levels of FcγRIIB expression on B cells vary as much as six fold between individuals. The factors that influence the expression of FcγRIIB on B cells among healthy donors are largely unknown. It has been reported that IL-4 reduces the expression of FcγRIIB on B lymphocytes and may play a role in Th2 response in mice [28]. It would be of great interest to determine the intrinsic and extrinsic factors that modulate the expression of FcγRIIB as these may hold the keys to treatment of autoimmune diseases. In light of the findings of Xiang *et al.* [13] and those presented here, selective modulation of FcγRIIB expression on PCs may represent a new therapeutic approach to antibody-mediated autoimmune diseases as previously suggested [29]. One important caveat is that neither T cells nor NK cells express FcγRIIB and this gives an advantage to modulate humoral immunity through FcγRIIB with little or no impact on the cellular immunity. Lastly, our findings that FcγRIIB functions independently of the BCR to eliminate PCs and inhibit naïve B cell activation while preserve the long-lived memory B cells supports its safe clinical use not only in targeting B cells for the treatment of autoimmune diseases, e.g. SLE, but also in suppressing autoantibody-mediated destruction to tissues or cells by intravenous immunoglobulin therapy (IVIg), e.g. immune thrombocytopenic purpura. Under these conditions, administration of either targeting mAbs or polyclonal gamma globulins acutely introduces a high level of antibodies in circulation. Despite the presence of a risk to inadvertently induce antigen-independent inhibition by FcγRIIB, memory B cells can be selectively preserved for replenishment to maintain B cell homeostasis. In addition, patients with systemic lupus erythematosus down-regulate the surface expression of FcγRIIB on memory B cells and PCs, leading to uninhibited expansion of B cells and autoantibody production [5, 6]. Our data strongly support a crucial role of FcγRIIB to function in the elimination of autoreactive B cells to prevent from antibody-mediated autoimmune diseases and further underscore the importance of antigen-independent inhibition by FcγRIIB as a peripheral checkpoint in human B cells. Namely, lower expression of FcγRIIB not only allows autoreactive PCs to break tolerance but also lowers the threshold for them to activate and expand, thereby allowing secretion and accumulation of more autoantibodies to form ICs that can deposit in tissues, e.g. the kidney, to cause damage and inflammation. It is then conceivable that restoration of the expression level of FcγRIIB on B cells may be beneficial for patients with SLE to alleviate lupus nephritis in that circulating ICs can then self-eliminate autoreactive PCs through apoptosis. Indeed, partial restoration of the expression level of ~40 % of FcγRIIB on B cells by retroviral transfection is sufficient to restore tolerance and ameliorate disease activity in lupus mice [30]. Since PCs are most sensitive to apoptosis triggered by FcγRIIB (Fig. 7) [13, 14] targeting FcγRIIB likely will affect PCs effectively, including those resident in the bone marrow.

## Conclusion

These results provide evidence that human peripheral blood PCs and naïve B cells but not memory B cells are main targets of FcγRIIB’s antigen-independent regulation. This observation suggests a mechanism by which antibody levels can be acutely regulated by ICs during antibody responses while maintaining B cell homeostasis. Moreover, our findings indicate that the graded expression levels of FcγRIIB in B cell subpopulations (PCs > memory cells > naïve cells) dictate the outcomes of FcγRIIB-mediated inhibition independent of BCR and provide new insights into safe use of antibody targeted therapy in autoimmune diseases, e.g. SLE. More importantly, we demonstrate for the first time that antigen-independent inhibition by FcγRIIB is mediated by Btk and p38 MAPK in human B cells. These findings in humans also extend our previous work on mouse B cells and further underscore the importance of antigen-independent FcγRIIB regulation of antibody responses in humans since a failure of FcγRIIB to mediate apoptosis of PCs might result in susceptibility to systemic lupus erythematosus [5, 6]. Taken together, FcγRIIB may be considered as a new drug target for selective modulation in the treatment of antibody-mediated autoimmune diseases.

## Abbreviations

ASCs: Antibody-secreting cells
BCR: B-cell receptor
ICs: Immune complexes
IL: Interleukin
mAb: Monoclonal antibody
PAP: Peroxidase-anti-peroxidase
PCs: Plasma cells
PWM: Pokeweed mitogen
sCD40L: Soluble CD40 ligand
SAC: *Staphylococcus aureus* Cowan
SLE: Systemic lupus erythematosus
Th2: T helper type 2
TLR: Toll-like receptor
